# The Impact of Body Mass Index and Medical Conditions on Home-based Anal Self-Sampling

**DOI:** 10.21203/rs.3.rs-2748914/v1

**Published:** 2023-03-31

**Authors:** Jenna Nitkowski, Maria E. Fernandez, Tim Ridolfi, Elizabeth Chiao, Anna R. Giuliano, Vanessa Schick, Michael D. Swartz, Jennifer S. Smith, Alan G. Nyitray

**Affiliations:** Medical College of Wisconsin; The University of Texas Health Science Center at Houston School of Public Health; Medical College of Wisconsin; The University of Texas; Moffitt Cancer Center and Research Institute; The University of Texas Health Science Center at Houston School of Public Health; The University of Texas Health Science Center at Houston School of Public Health; University of North Carolina at Chapel Hill; Medical College of Wisconsin

**Keywords:** body mass index (BMI), obesity, physical disability, self-sampling, human papillomavirus (HPV), anal cancer, screening

## Abstract

**Purpose.:**

Self-sampling is increasingly being used in screening programs, yet no studies to date have examined the impact of bodily characteristics on self-sampling experiences. Our objective was to assess whether body mass index (BMI) and physical disability were associated with anal self-sampling difficulty.

**Methods.:**

We recruited sexual minority men (SMM) and trans persons in Milwaukee, Wisconsin to participate in an anal cancer screening study. Between January 2020 and August 2022, 240 participants were randomized to a home (n=120) or clinic (n=120) screening arm. Home participants received a mailed at-home anal self-sampling kit and were asked to attend a baseline clinic visit where biometric measurements were collected. Participants were asked to complete a survey about their experience with the kit. This research utilizes data from participants who used the kit and completed a baseline clinic visit and post-swab survey (n=82). We assessed the impact of BMI and physical disability on reported body or swab positioning difficulty.

**Results.:**

Most participants reported no or little difficulty with body positioning (90.3%) or swab positioning (82.9%). Higher BMI was significantly associated with greater reported difficulty with body positioning (aOR=1.10, 95% CI 1.003–1.20, *p*=.04) and swab positioning (aOR=1.11, 95% CI 1.02–1.20, *p*=.01). Physical disability was not significantly associated with body or swab positioning difficulty. Specimen adequacy did not differ by BMI category (*p*=.76) or physical disability (*p*=.88).

**Conclusion.:**

Anal self-sampling may be a viable option to reach obese persons who may be more likely to avoid screening due to weight-related barriers.

## Introduction

Anal cancer is typically diagnosed in the early 60s [1], with disproportionate incidence among sexual minority men (SMM).[2] Although currently there are no official guidelines for anal cancer screening, the United States Preventative Task Force issued a draft research plan in December 2022 for development of an anal cancer screening recommendation.[3] Self-sampling as an anal cancer screening method is being studied and has demonstrated high acceptability among SMM and persons living with HIV.[4–6] Since 90% of anal cancers are associated with human papillomavirus (HPV) infection,[2] HPV anal canal self-sampling is a potential method to assess risk for anal cancer. However, self-sampling may be difficult for older adults who may be more likely to have age-related physical disabilities such as arthritis or chronic lower back pain. It is also unknown how other bodily characteristics may affect self-sampling. For example, obesity could present challenges related to obtaining a sample due to issues with mobility or pain. Obesity in the United States is rising, with an estimated prevalence of 42% in 2017–2020,[7] and nearly one in four adults over the age of 51 are obese.[8]

Cervical cancer research has shown that obesity is associated with lower likelihood of attending cervical cancer screening and higher likelihood of inadequate smears collected by a clinician.[9] However, this research has only looked at in-person clinic screening. No studies to our knowledge have examined the effect of obesity or physical disability on self-sampling, even cervicovaginal self-sampling which is increasingly being utilized in cervical cancer screening programs across the world.[10] Home-based self-sampling could be a potentially viable option to reach obese persons who are less likely to attend screening. However, research is needed to investigate the impact of bodily characteristics and physical disability on self-sampling experiences and specimen adequacy.

## Methods

Data for this research come from the Prevent Anal Cancer Self-Swab Study in Milwaukee, Wisconsin, USA. The study protocol has been described in detail elsewhere.[11] Study activities were approved by the Medical College of Wisconsin Human Protections Committee (protocol # PRO00032999). Eligible participants were 25 years of age or older and reported sex with men in the last five years or identified as gay or bisexual. After providing informed consent, participants were randomized to either a home or clinic arm. A total of 240 participants were randomized between January 2020 and August 2022 (home = 120, clinic = 120). Due to the COVID-19 pandemic, study activities were paused between March 2020 and November 2020. The following research uses data from the home-based arm, where participants received a mailed at-home anal HPV self-sampling kit.

Each kit contained a flocked swab (COPAN Italia s.p.a.), standard transport media (Qiagen) pre-labeled with a unique ID, illustrated self-sampling instructions, a biohazard bag, return packaging, and sample return instructions. Instructions were written at a sixth-grade reading level in English or Spanish. After completing the at-home anal self-sampling, home-based participants were asked to complete a post-swab survey about their experience. They were also asked to make a clinic visit where they received a clinician-collected anal swab. This research utilizes data from participants who used the kit, and then completed a post-swab survey and baseline clinic visit (n = 82).

## Measures

### Outcomes.

We investigated two outcomes in this research assessing body and swab positioning difficulty. Both questions were asked during the post-swab survey. Body positioning difficulty was coded as a binary variable (yes/no). After completing the kit, participants were asked *“Was it hard to position your body to insert the swab into your anus?*”, with response options of “*No, it wasn’t hard*”, “*It was a little hard*”, “*It was moderately hard*”, or “*It was very hard*”. Difficulty with anal self-sampling was coded as “yes” if a participant indicated it was moderately or very hard, while responses of no or a little hard were classified as no difficulty with anal self-sampling. Swab positioning difficulty was measured by the post-swab survey question “*Was it hard to position the swab at the opening to your anus?*”, with response options of “yes” or “no”.

### Exposures.

Height and weight measurements were completed at the clinic visit and recorded by study personnel. Body mass index (BMI) was calculated as weight (kg)/height (m^2^).[12] Physical disability was assessed in the baseline survey. Participants were presented with the question “*Here is a list of medical conditions that may make it harder to use the swab. Has a doctor ever said that you have any of the following (check all that apply)?*” and given response options such as arthritis, carpal tunnel syndrome, and chronic lower back pain. We combined responses into a composite variable representing presence or absence of any physical disability (yes/no). Participant demographic characteristics such as age, race/ethnicity, gender identity, sexual orientation, education, and HIV status were asked during the eligibility and baseline survey.

## Statistical analyses

Descriptive statistics of participant demographic characteristics, biometric measurements, and physical disability were conducted. Multivariable logistic regression analyses were conducted between the primary exposures (BMI and physical disability) and reported difficulty with body and swab positioning for self-collection. Age was included as a potential confounder. Firth’s penalized likelihood estimation was used for the multivariable logistic regression analysis examining body positioning difficulty to account for unequal variances in the outcome variable. Adjusted odds ratios are reported with 95% confidence intervals. All statistical analyses were conducted in SPSS version 28.[13]

## Results

Sample participants ranged in age from 25.7 to 78 years old, with a mean age of 46.2 years old (Table 1). Over one-third of participants (36.6%) were age 55 and older. Participants identified as non-Hispanic White (63.0%), non-Hispanic Black (24.7%), and Hispanic or Latino/x (12.3%). A total of 96.3% of participants identified as a man and 3.7% identified as trans or non-binary. Most participants identified as gay (86.6%) or bisexual (11.0%). The majority of participants (68.3%) had 16 or more years of education. A total of 28.0% of participants were living with HIV.

Participant height in this sample ranged from 163.3 to 194.2 centimeters, with an average of 177.6 centimeters (Table 1). The average weight was 96.3 kilograms and ranged from 55.4 to 167.8 kilograms. Average body mass index was 30.5 which is classified as obese and ranged from 18.3 to 55.4. Approximately one in five participants (20.7%) were classified as a “healthy weight”, while 34.1% and 43.9% were classified as overweight and obese, respectively. Approximately 15% of sample participants reported that their doctor had ever said they had a medical condition hypothesized to make it harder to use the swab. Specifically, arthritis (n = 9), carpal tunnel syndrome (n = 4), cerebral palsy (n = 1), chronic lower back pain (n = 6), motor neuron disease (n = 1), movement disorder (n = 2), multiple sclerosis (n = 1), spina bifida (n = 1), or spinal cord injury (n = 1) were reported.

When asked if it was hard to position the body to insert the swab into the anus, most participant responses were “no” (61.0%) or “a little” hard (29.3%), while 9.7% of participants reported that it was “moderately” or “very” hard. Approximately 17% of sample participants reported that it was hard to position the swab at the opening to the anus.

Table 2 shows the results of multivariable logistic regression analyses. Body mass index had a significant positive association with difficulty positioning the body to insert swab into anus (aOR 1.10, 95% CI 1.003–1.20, *p* = 0.04). For a one-unit increase in BMI score, there was a 10% increase in the odds of reporting difficulty with body positioning while controlling for age and physical disability. Body mass index was also significantly associated with swab positioning difficulty (aOR 1.11, 95% CI 1.02–1.20, *p* = .01). Age and physical disability did not have significant associations with either body or swab positioning difficulty.

Most swabs completed at home were adequate for HPV genotyping, regardless of BMI or physical disability. There was no significant difference in specimen adequacy by body mass index categories (*p* = 0.76) although obese participants had the highest proportion of adequate specimens (94.4%), followed by overweight participants (92.9%), and under/healthy weight participants (88.9%). All specimens collected by participants who reported difficulty with body positioning (n = 8) were adequate for HPV genotyping. Similarly, specimen adequacy did not significantly differ by physical disability status. Specimen adequacy was 91.7% for participants reporting a physical disability and 92.9% for those without a physical disability.

## Discussion

This is the first study to our knowledge to examine the relationship between body mass index, physical disability, and self-sampling. Research on self-sampling shows that persons find it acceptable, but studies are lacking on whether bodily characteristics affect self-sampling experience or specimen adequacy. While most participants in this study reported no or little difficulty, greater body mass index was associated with greater difficulty with body and swab positioning. Despite these difficulties, there was no difference in specimen adequacy by body mass index category. Similarly, specimen adequacy did not differ by whether a participant reported a physical disability. All participants who reported that it was moderately or very hard to position their body to insert the swab into the anus had adequate specimens.

There are a few limitations to note. Sample participants self-selected into this randomized clinical trial about anal cancer screening and engaged in baseline study activities. Therefore, their anal self-sampling experiences may not be representative of the overall population of sexual minority men. Another limitation is the small number of reported physical disability cases which may have limited the power to detect an association with reported body or swab positioning difficulty. Finally, participants who found it more difficult to position their body may have also been more likely to inadvertently sample the perianal region and thus present a perianal specimen that was adequate rather than an anal canal specimen.

Research on obesity and gynecological cancer screening has shown that obese women are less likely to screen and report weight-related barriers as reasons for delaying screening.[14] These barriers are largely due to in-person clinic visits, such as unwanted weight-loss lectures by providers or too-small gowns, tables, and equipment in clinics.[14] Our findings showed that overweight and obese participants completed baseline study activities and were able to collect an adequate anal self-sample at home. This suggests that home-based self-sampling may be a potentially viable method to reach populations less likely to screen due to in-person barriers, such as those reported by obese patients.

## Conclusion

This study is the first to examine the association between bodily characteristics and self-sampling. Most participants reported no or little difficulty positioning their body to insert the swab into their anus. While body mass index was significantly associated with greater difficulty positioning the body and swab, there was no difference in specimen adequacy by body mass index category or physical disability. Self-sampling can be a potential method to reach overweight and obese persons who may be more likely to delay screening due to weight-related barriers.

## Figures and Tables

**Table 1:** Characteristics of home-based participants who used a PAC Pack and completed a post-swab survey and baseline clinic visit in the Prevent Anal Cancer Self-Swab Study, 2020–2022, Milwaukee, Wisconsin, USA (n = 82).

	n (%) or Mean (SD), Range
*Exposures*	
**Age, years**	46.2 (13.7), 25.7–78.0
**Race/ethnicity**	
Non-Hispanic White	51 (63.0)
Non-Hispanic Black	20 (24.7)
Hispanic or Latino	10 (12.3)
Missing	1
**Gender identity**	
Man	79 (96.3)
Trans or non-binary	3 (3.7)
**Sexual orientation**	
Gay	71 (86.6)
Bisexual or queer	11 (13.4)
**Education**	
12 years	5 (6.1)
13–15 years	21 (25.6)
16 years	20 (24.4)
More than 16 years	36 (43.9)
**HIV status**	
Positive	23 (28.0)
Negative	59 (72.0)
**Height, cm**	177.6 (6.3), 163.3–194.2
**Weight, kg**	96.3 (24.3), 55.4–167.8
**Body mass index (BMI)**	30.5 (7.3), 18.3–55.4
**BMI**	
Underweight (< 18.5)	1 (1.2)
Healthy weight (18.5–24.9)	17 (20.7)
Overweight (25.0–29.9)	28 (34.1)
Obese (≥ 30.0)	36 (43.9)
**Ever had a physical disability**	
Yes	12 (14.6)
No	70 (85.4)
*Outcomes*	
**Was it hard to position your body to insert the swab into your anus?**	
No, it wasn’t hard	50 (61.0)
It was a little hard	24 (29.3)
It was moderately hard	6 (7.3)
It was very hard	2 (2.4)
**Was it hard to position the swab at the opening to your anus?**	
Yes	14 (17.1)
No	68 (82.9)

**Table 2:** Multivariable regression analyses examining the association between physical characteristics and difficulty with body or swab positioning in the Prevent Anal Cancer Study, 2020–2022, Milwaukee, Wisconsin, USA (n = 82).

	Body positioning difficulty	Swab positioning difficulty
	aOR (95% CI)	aOR (95% CI)
**Age, years**	1.00 (.95–1.06)	1.02 (.97–1.07)
**Body mass index (BMI)**	1.10 (1.003–1.20)*	1.11 (1.02–1.20)*
**Ever had a physical disability** ^1^		
Yes	2.79 (.45–17.34)	1.81 (.35–9.33)
No	1.0	1.0

Note: aOR = adjusted odds ratio.

* *p* < .05

1 Physical disability = arthritis, carpal tunnel syndrome, cerebral palsy, chronic lower back pain, motor neuron diseases, movement disorders, multiple sclerosis, spina bifida, or spinal cord injury.

## Data Availability

The datasets generated during the current study are not publicly available.
